# Hygiene During Childbirth: An Observational Study to Understand Infection Risk in Healthcare Facilities in Kogi and Ebonyi States, Nigeria

**DOI:** 10.3390/ijerph16071301

**Published:** 2019-04-11

**Authors:** Helen Buxton, Erin Flynn, Olutunde Oluyinka, Oliver Cumming, Joanna Esteves Mills, Tess Shiras, Stephen Sara, Robert Dreibelbis

**Affiliations:** 1 Disease Control Department, London School of Hygiene and Tropical Medicine, London WC1E 7HT, UK; Helen.Buxton1@lshtm.ac.uk (H.B.); Oliver.Cumming@lshtm.ac.uk (O.C.); Joanna.EstevesMills@lshtm.ac.uk (J.E.M.); tess.shiras@gmail.com (T.S.); 2 Infection and Immunity, South Australian Health and Medical Research Institute, Adelaide 5000, Australia; Erin.Flynn@sahmri.com; 3 Maternal and Child Survival Program (MCSP)/Save the Children Nigeria, Abuja, Nigeria; olutundemma@gmail.com; 4 Maternal and Child Survival Program (MCSP)/Save the Children US, Washington, DC 20036, USA; ssara@savechildren.org

**Keywords:** labour, child-birth, maternal infection, neonatal infection, infection prevention and control, hand hygiene

## Abstract

**Background**: Infections acquired during labour and delivery are a significant cause of maternal and child morbidity and mortality. Adherence to hand hygiene protocols is a critical component of infection prevention strategies, but few studies have closely examined the hand hygiene of health care providers with sufficient detail to understand infection risks and prioritize prevention strategies. **Methods**: This observational study was conducted in six healthcare facilities in Nigeria. In each, five women were observed from the onset of labour through to delivery of the placenta. Hand hygiene infection risk was estimated for all procedures requiring aseptic technique compared against adherence to proper hand hygiene protocol and potential recontamination events. **Results**: Hands were washed with soap and sterile gloves applied with no observed recontamination before only 3% of all observed procedures requiring aseptic technique. There was no significant difference in hygiene compliance between midwives and doctors nor facilities or states. Adherence to proper hygiene protocol was observed more in morning compared to afternoon and night shifts. **Conclusions**: This study highlights that hand hygiene remains a barrier to delivering high-quality and safe care in health facilities. Improving hygiene practices during labour and delivery will require strategies that extend beyond infrastructure provision.

## 1. Introduction

Sepsis is a key contributor to both maternal and neonatal mortality, accounting for 15% of all neonatal deaths [1] and 1 in every 10 maternal deaths [2]. Maternal sepsis is defined as a life-threatening organ dysfunction resulting from infection during pregnancy, childbirth, post-abortion, or postpartum period [3]. Neonatal sepsis, particularly early onset neonatal sepsis (within the first 7 days of life), is an infection in the blood of the newborn transmitted from mother to child during childbirth or in the care-giving environment after birth [4,5]. 

Although the evidence is limited or of low quality, techniques to ensure births are clean and hygienic have been associated with decreases in all-cause neonatal mortality, infection-related mortality, and infections of the umbilical cord [6]. The hand hygiene of healthcare workers (HCWs) is the cornerstone of these practices. 

Quality of care assessments have demonstrated that compliance with hand hygiene protocols is often far lower than for other evidence-based quality of care interventions [7]. Poor compliance with hand hygiene protocols is often strongly associated with structural barriers, including overcrowding, high patient loads and understaffing [8], limited time [9] and inadequate infrastructure [10]. Gaps in institutional culture such as a lack of institutional guidelines [11,12], a lack of compliance by superiors [11] and widespread use of gloves alone for hand hygiene [8] have also been cited as barriers to hand hygiene. More complex patient care processes such as labour and delivery which require a greater frequency of hand hygiene actions than other patient care procedures (e.g., canula insertion) are associated with an increased reduction in adherence to hand hygiene protocol [13].

The WHO-recommended hand hygiene protocol for HCWs centres around “five moments”: before touching a patient; before an aseptic/clean procedure; after exposure to bodily fluids; after touching a patient; and after touching a patient’s surroundings [13]. Childbirth constitutes multiple overlapping procedures and exposures over an indefinite period of time, often resulting in more than one “moment” presenting simultaneously. However, studies that focus on hygiene compliance often reduce assessments to a dyadic relationship between hygiene actions and individual procedures, such as handwashing with soap (HWWS) and glove use prior to preparing for the birth [14]. These assessment methods do not capture the shifting dynamics of risk inherent in childbirth.

The data presented here are part of a larger mixed-methods investigation of clean birthing practices in healthcare facilities (HCFs) in Ebonyi and Kogi states, Nigeria, with a focus on the role of water, sanitation, and hygiene (WASH) in the prevention of maternal and newborn infections and sepsis. According to recently available global monitoring data, approximately half of HCFs in Nigeria have access to an improved water source on premises and clean, usable sanitation facilities; 63% of facilities have handwashing facilities at the point of care; and 43% of facilities appropriately segregate, treat, and dispose of waste [15]. Nigeria has the fourth-highest maternal mortality rate in the world, at 814 deaths per 100,000 births [16], over 10 times that of the global SDG target of 70 [17]. At 34 deaths per 1000 live births [1], neonatal mortality rates in Nigeria are almost three times the global SDG target of 12 per 1000 [17]. Three quarters of these neonatal deaths are estimated to occur in the first week of life [18]. Low WASH coverage likely contributes to high rates of maternal and neonatal mortality in Nigeria.

Objectives were to document current infection prevention and control (IPC) strategies at HCFs and observed hygiene practices along the continuum of care from childbirth to the home environment. This manuscript focuses specifically on clean birthing practices during labour and delivery in a sample of 31 births observed in six HCFs. Findings from the post-natal and home observations are presented in a follow-on publication. 

## 2. Materials and Methods

### 2.1. Study Setting

This observational study was conducted in two states in Nigeria (i.e., Ebonyi and Kogi) over 4 weeks in July 2017. Based on a 2013 assessment, 60% of births in Ebonyi and 79% in Kogi occur at an HCF [19], and neonatal mortality rates stand at 37/1000 births in Ebonyi and 35/1000 births in Kogi [19]. 

**Figure 1:**
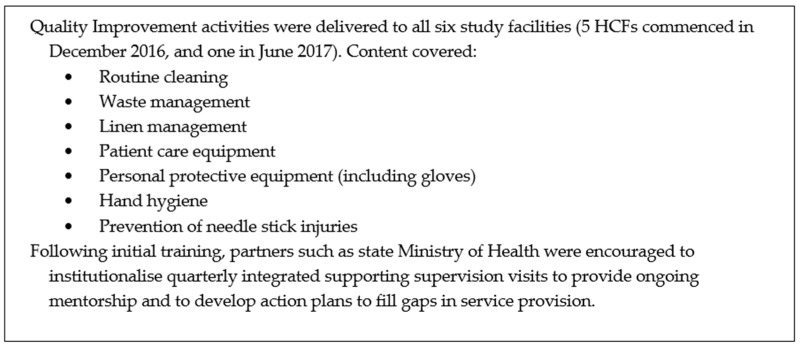
Details of Maternal and Child Survival Program (MCSP)-support quality improvement (QI) programming. HCF: healthcare facility.

All facilities included in the study received support from the United States Agency for International Development (USAID)-funded Maternal and Child Survival Program (MCSP) to improve quality of care. MCSP/Nigeria launched in 2014 and focused on strengthening the national-level Maternal Newborn and Child Health (MNCH) policy and improving the quality and utilization of services. MCSP provided facility-based training on basic and comprehensive obstetric and newborn care, essential newborn care, and quality of care. Time dedicated to hand hygiene training was limited during the training programme. For example, it constituted just 10 min of the essential newborn care course and did not include skill practice. More specific quality of care improvements are detailed in Figure 1. 

Promotion of hand hygiene, including the use of alcohol-based hand sanitisers, and clean delivery practices were integrated into trainings. Hand hygiene training materials focused predominantly on techniques for handwashing, but included only brief mention of when to wash hands—the WHO concept of “five moments” was not integrated into training materials. Further details on the MCSP program are included in Supplementary Materials (Supplemental Information 1).

### 2.2. Study Sample

As a descriptive exploratory study, sample size was based primarily on resources availability. Three facilities were selected per state, representing one primary, one secondary and one tertiary HCF. In each state, facilities with the highest number of monthly deliveries were purposively selected to ensure enough births would be observed in the duration of the study. Women were eligible for enrolment if they presented at the HCF for delivery prior to entering the second stage of active labour and were not undergoing excess pain or concern during the consent process. Women with conditions associated with increased risk of complications during delivery (e.g., high blood pressure, diabetes or pre-term labour) were excluded from enrolment. If complications arose during delivery, observations were suspended. Women under the age of 18 were also excluded from the study. Any woman who met inclusion criteria, presented at the HCF to deliver, and gave consent, was invited to participate, up to a total of five consenting women per facility. The labour and delivery observation period commenced once a woman was admitted to deliver in the delivery unit and terminated once she delivered the placenta. Observations of the post-natal period and qualitative data will be reported in a follow-on publication. 

### 2.3. Data Collection

Structured facility assessments adapted from existing tools (WHO WASHFIT [20] and SoapBox WASH & Clean Toolkit [21]) were completed in all facilities prior to data collection. These included a structured facility observational checklist (walk-through) that included information on hygiene infrastructure and supplies at multiple locations in the health care facility and facility staffing. A facility needs assessment survey was completed in interview format with the officer-in-charge of the maternity ward. Further details on the tools and key findings are reported in a forthcoming publication. Birth observations were conducted by qualified midwives. A standardized direct observation tool was developed based on a tool used in a previous quality of care study [22] and iteratively refined in collaboration with study midwives over a 7-day pre-test period in HCFs in Abuja. All data collectors received 7 days of training, including simulated birth observation practice and field practice in non-study HCFs in Ebonyi state. Inter-rater reliability was monitored throughout the training period and data collection did not commence until observation results were consistent. Training also included emergency protocols for instances where midwives observed behaviours or situations that placed the woman or the baby at risk, specifically: immediately halting the observation and reporting to facility head or intervening in life-threatening situations.

The study midwives worked on a shift rotation to ensure the continuous availability of data collection staff. Observations began when consenting women were clinically confirmed by the attending midwife or doctor to be in active labour (cervical dilation > 3 cm) and admitted to delivery at the facility. Observation periods reported here covered labour and delivery (until placenta was delivered).

Data collectors explained to the women that the decision to participate would have no bearing on their care, and participants were given regular breaks in observation and could request to break or terminate observation at any point. Informed consent was also collected from all staff at the start of every shift during the observation period. Data were collected on a pre-programmed digital platform (SurveyCTO software. Dobility, Inc. Cambridge, MA, USA). 

### 2.4. Data Analysis

Data analysis and management was done in Stata SE v15 (Stata Corp, College Station, TX, USA). Facility-level data were examined descriptively and used to provide context for observation data. Data from qualitative text entries were reviewed and, where applicable, recoded into structured observation format. 

Our analysis positions hand hygiene protocol as a continuum of actions situated within the fluid context of the delivery room. This is in recognition that hand hygiene protocol during patient care requires HCWs to be both proactive and reactive—a HCW must take action to ensure hand hygiene is adequate prior to patient contact and prior to procedures; and the HCW must react to potential exposures to contamination, such as contact with another patient or contact with bodily fluids, by HWWS and changing gloves [13]. Data were described dynamically through an analysis process that followed specific actors—individual midwives, doctors, or other HCF staff—through their entire sequence of observed behaviours during labour and delivery, including but not limited to: medical procedures on the woman, interactions with other patients, contact with equipment or objects, and any hygiene actions. 

For all actors, time-specific hygiene scores were calculated that reflected both hand hygiene actions taken by the HCW and any potential contamination or recontamination of hands. We identified five categories of hand hygiene (see Table 1). Categories 1 through 3 detailed specific hand hygiene practices. Categories 4 and 5 detailed potential observed contamination. Category 4 equates to no hygiene action taken in reaction to any observed event that would invalidate the aseptic technique required for the procedure under review (e.g., non-invasive contact with the woman or the woman’s surroundings). Category 5 equates to no hygiene action taken following high-risk exposures aligned with WHO criteria [13]: contact with another patient; contact with bodily fluids; contact with mucous membranes, or contact with clinical waste or faeces. Simply put, Category 1 can be considered the most hygienic and Category 5 the least hygienic state of the health worker’s hands. We did not include use of alcohol-based hand rub (ABHR) within our categories, as our data indicates that ABHR was not used during study observations. Because our categories were time-specific, actors changed classification throughout the observation period based on hand hygiene actions taken and any potential exposure that re-contaminated hands or invalidated the aseptic technique.

The focus of the analysis was patient protection from pathogen transmission; therefore, we identified all procedures conducted by HCWs which require aseptic technique—defined here as requiring HWWS/alcohol rub, sterile gloves to be worn, and that gloved hands have no contact with nonsterile surfaces prior to the procedure. Specific procedures of interest were: vaginal examinations, insertion of urine catheter, insertion of IV cannula, artificial rupture of membranes, manual removal of placenta, manual removal of blood clots, cord tie/clamping, and cord stump contact. For each observed procedure, the time-specific hygiene category for the actor that conducted the procedures was noted. 

Descriptive statistics were used to analyse the proportion of aseptic events that were conducted within each of the five risk categories. To explore associations between specific factors and compliance with hygiene protocol, hygiene categories were simplified to a three-point scoring system: 1) Compliant (Category 1), Inadequate (Categories 2 and 3), and Risky (Categories 4 and 5). Somers’ D, a non-parametric alternative to the chi square test that adjusts for clustering, was used to assess the probability of an observed event having an improved hygiene score by provider type (doctor vs. midwife), HCF type (primary vs. secondary vs. tertiary), shift (morning vs. afternoon vs. night), and state (Ebonyi vs. Kogi). All analyses were adjusted for repeated observations of the same provider within each observation period. 

### 2.5. Ethics

Ethics approval was granted by the Institutional Review Board at London School of Hygiene and Tropical Medicine (13643), and the Ethical Review Boards of Kogi state (MOH/KGS/1376/1/84) and Ebonyi state (SMOH/ERC/33/017). 

## 3. Results

### 3.1. Delivery Unit Conditions

The average number of deliveries per study facility was 131 per month (range: 40–216 per month); this ranged from 49 per month in primary facilities to 125 in the secondary and tertiary facilities. All six facilities had a functioning handwashing facility (HWF) with soap and water in the delivery unit at the time of the walk-through inspection, however during 1 of the 31 observed births soap was not available during delivery. All units had a sink with a connected tap available in the delivery unit, but two facilities used Veronica buckets (a bucket with a tap and basin beneath it) because of broken taps. Water points were accessible in all six delivery units, located close to point of care and not cluttered with any items other than those for washing hands. There were no disposable towels present at any of the HWFs nor any hand hygiene posters. 

### 3.2. Labour and Delivery Observations

In total, 31 pregnant women across the six HCFs were recruited for the study. The average duration of each observation was 256 min (range: 61–745 min). Facility-provided gloves were available in the delivery room in all but one of the observed deliveries. On average, there were five different actors present during labour and delivery, including doctors, midwives and hospital auxiliary staff. Observations recorded an average of three handwashing events per delivery (range: 1–12); glove changes were observed twice as often (mean: 7, range: 1–27). Alcohol-based hand sanitizers were available in four out of six facilities, but were not used at any time during observations.

We recorded a total of 201 procedures on the observed women which required aseptic technique—an average of 6 per birth. Vaginal examinations (VEs) were the most frequent procedure; observed an average of four times per patient (range 1–10). The mean time span between observed VEs on an individual woman was 79 min (range: 28–248 min). Insertion of a urine catheter was observed during all 31 observations. Artificial rupture of the membranes was observed in more than half of the observations (17/31); and manual removal of the placenta or blood clots, and suturing of the perineum were observed in just over a third (11/31). Specific to the newborn, there were 51 instances of observed cord contact during observation periods, slightly less than two times per observation.

Overall, only 3% of all observed events requiring aseptic technique were conducted in Category 1 where hands had been washed with soap and sterile gloves applied with no observed recontamination of the hands (Table 2). The majority were conducted when no hand hygiene action had been taken following potential recontamination of hands/gloves through lesser or higher risk exposures (Category 4: 41% of observed procedures requiring aseptic technique; Category 5: 27% of observed procedures requiring aseptic technique). 

Distribution among hygiene categories differed when procedures were stratified by those conducted on the woman and those conducted on the newborn; 94% of neonatal procedures requiring aseptic technique were conducted in Category 4 or Category 5 compared to 59% of maternal procedures requiring aseptic technique. While 4% of maternal procedures were conducted in Category 1—HWWS and gloves changed—no procedures conducted on the newborn were completed following full hygiene protocols (See Table 3).

#### 3.2.1. Hygiene Risk during Mother-Specific Procedures

**Figure 2:**
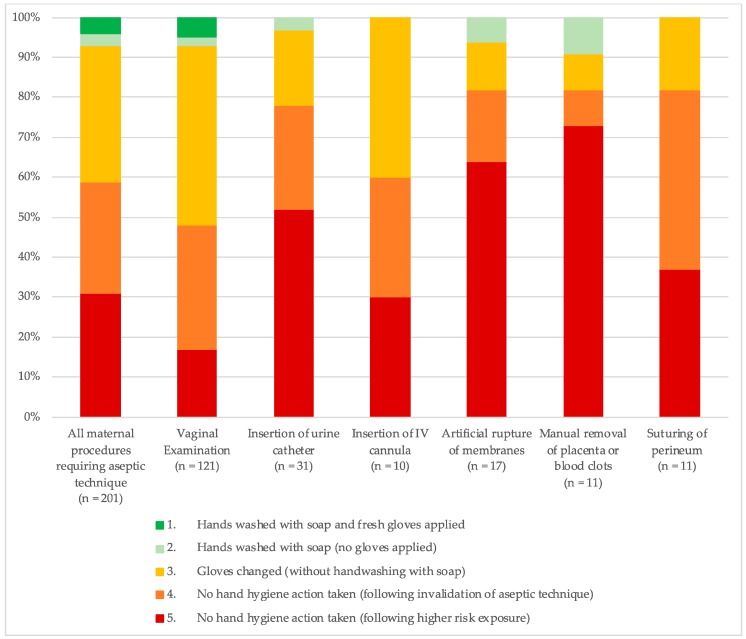
Hygiene categories during individual mother-specific procedures requiring aseptic technique. VE: vaginal examination.

Among procedures conducted on women, adherence to hygiene protocol varied by procedure (Figure 2). The procedure with the highest adherence to hand hygiene protocol was VE. However, only 6% of VEs were undertaken when hands had been washed with soap and new gloves applied (Category 1), and 45% of observed VEs were conducted following glove changes but without intermediary hand washing with soap (Category 3). In contrast, 65% of observed procedures to artificially rupture the membrane and 74% of procedures to manually remove the placenta or blood clots were conducted following a high-risk potential contamination event (Category 5). All observed procedures to suture the perineum (*n* = 11) were conducted following invalidation of the aseptic technique, in either Category 4 (45%) or Category 5 (37%). See Supplementary Materials Table S1 for more details.

There was no significant difference in hygiene compliance comparing nurse/midwives to doctors or comparing facility type (Table 2). Compliance did not differ significantly by state. However, both afternoon and night shifts were associated with reduced likelihood of improved hygiene practices compared to morning shifts (afternoon: Somers’ D = −0.2, *p* = 0.04; night: Somers’ D = −0.2, *p* = 0.008). 

#### 3.2.2. Hygiene Risk during Neonate-Specific Procedures

**Figure 3:**
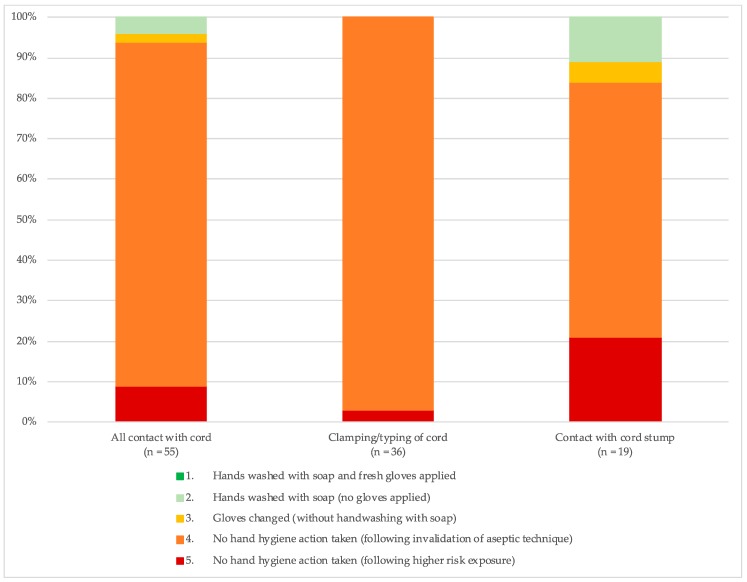
Hand hygiene during neonate-specific procedures requiring aseptic technique during labour and delivery.

Figure 3 shows the hygiene category for procedures on newborns. All observations of cord clamping/tying prior to cutting the cord were classified as Category 4 or 5. The majority of contact with the cord stump following cord cutting (63%) were also conducted in Category 4. See Supplementary Materials Table S2 for more details.

## 4. Discussion

Findings from this study suggest that hand hygiene remains inadequate despite previous facility-level training on hand hygiene protocols and availability of handwashing materials at convenient locations in delivery units. We found that the vast majority of procedures requiring aseptic technique on both the woman and the newborn were conducted without adequate hand hygiene, although findings suggest that procedures conducted on the newborn had lower rates of compliance with aseptic techniques. Low rates of hygiene compliance were observed among all providers (nurses, midwives, doctors), at all facility types, and across both study sites. We observed a reduced likelihood of adherence to hygiene protocol in both evening and afternoon shifts compared to the morning shifts.

This is consistent with previous studies that have documented low levels of hand hygiene compliance in health care settings in both low and high income settings [13,23]. Previous observational studies have measured adherence to hand hygiene protocol by monitoring if HCWs washed hands prior to and after procedures and have found compliance is much higher following a procedure rather than prior [13,22]. Our approach provides a more nuanced picture of compliance by examining HCW response to invalidation of aseptic technique prior to and during procedures, as well as enabling comparison based on the type of procedure being undertaken. 

We observed more frequent VEs than recommended in WHO guidelines [24]. While VEs were associated with the highest level of compliance among all procedures requiring aseptic technique, hand hygiene compliance levels before VEs were still sub-optimal. VEs are used to assess progress in labour, and to provide early warning if progress is abnormally slow [25]. However, the procedure can also introduce infection to the uterus and the newborn if hand hygiene protocols are not adequately followed [25]. We also observed higher than expected frequency of invasive procedures on the woman that present significant infection risks, including artificial rupture of membranes in over half of our sample, manual removal of the placenta or blood clots in over one-third of women and urinary catheterisation conducted at least once in every observed delivery. These findings are explicit issues related to quality of care, which must be addressed to improve patient outcomes and prevent infection at the time of birth. 

This study is not intended to serve as an evaluation of MCSP-supported hygiene programming; we do not have information on pre-training conditions practices and our sample was not intended to be representative of all facilities supported by MCSP. However, our findings have important implications for the effectiveness of current strategies used for improving behavioural outcomes related to hand hygiene during labour and delivery. Many studies cite the lack of WASH infrastructure and soap as a barrier preventing the opportunity for hand hygiene [11,26]. Our data found there was physical opportunity for handwashing in all facilities. This suggests that improvements in physical opportunity alone are not sufficient to increase hand hygiene practices among HCWs. Theories of behaviour change position enactment of the repetitive act of hand hygiene in a busy health care setting is dependent upon responsiveness to external cues which trigger the behaviour at the appropriate moment [27,28]. Though the WHO-recommended “five moments” approach is explicitly crafted around the requirement to trigger behaviour, the training manual used in this intervention focused predominantly on technique rather than moments for hand hygiene. Updated training guidelines reference the WHO five moments [29], and we expect this to be further integrated into subsequent trainings. 

The high proportion of maternal procedures which were conducted in Category 3 (i.e., gloves are changed but hands are not washed in between) suggests that hand hygiene action is being triggered at the required moment but the target behaviour is incomplete. All current guidelines on glove use in HCFs [13,30] note that the act of donning and removing gloves can result in contamination when not coupled with HWWS. The prevalence of glove changes without intermediary HWWS may be a result of time pressure in the delivery room. Time pressure may also explain the association of night and afternoon shifts with reduced hand hygiene compliance, as these shifts had reduced personnel compared to morning shifts. Alcohol-based hand rubs (ABHRs) could provide a more expedient and efficient system for hand hygiene, particularly when changing gloves. ABHRs that are effective against many of the pathogens associated with maternal and neonatal infections [31] have been found to improve hand hygiene in high-income settings when accessible through mobile dispensers or at the point of care [32,33]. Recent innovations in response to widespread epidemics (e.g., Ebola virus disease) have called for hand hygiene guidelines to consider disinfection (via ABHR of gloved hands) for procedures on the same patient as an alternative to full hand hygiene protocol [34]. One study found that ABHR protocols resulted in a significant reduction in the incidence of late-onset infections in a neonatal intensive care unit [35]. However, ABHRs alone cannot be viewed as a substitute for soap and water, and hand hygiene is especially intended to remove blood and bodily fluids from hands. More research on practical solutions to enable simple and expedient hand hygiene in healthcare settings should be prioritised. Although ABHRs were available in the majority of births in our study, they were not used by HCF staff. As with handwashing infrastructure, provision alone is not sufficient to ensure adoption and use.

When compared to maternal procedures, contact with the newborn’s cord stump was more likely to be conducted without prior hand hygiene of any form. HCWs may group contact with the cord stump together with a larger sequence of activities to provide newborn care, rather than a distinct activity that requires a specific hygiene protocol. This could be addressed through more thorough training, yet integrated approaches that address hygiene and handwashing as one of multiple quality of care areas may not provide sufficient time to reinforce hygiene protocol, and thus may fail to translate into improvements in practice [28].

Our findings showed no difference in rates of compliance based on provider type (nurse/midwife vs. doctor), suggesting that non-compliance may be embedded in institutional norms. Disrupting these norms will require addressing gaps in provider motivation. Previous studies have positioned protection of the self as a more prevalent motivator for HCWs compliance with hand hygiene protocol than protection of the patient [22,36]. Intermediary HWWS reduces the risk of pathogen transmission to the patient, the very low proportion of procedures requiring aseptic technique which were conducted in Category 1—gloves changed and HWWS—may indicate that patient protection is not currently a prevalent driver of health care worker behaviour. Although we advocate for the adoption of the WHO five moments approach, we acknowledge that from a behavioural science perspective, this is still a highly medicalised approach which focuses predominantly on improving knowledge of disease risk and skills development [32]. Other approaches which prioritise the development of motives, habits and other non-cognitive drivers of health behaviour [32,33,34] have proven effective in community-based and school-based interventions. Our data suggest that there is a barrier in the translation of protocol to behaviour, and a wider-range of behavioural-science-informed interventions combined with the WHO five moments approach may go some way toward addressing this. 

This study has several limitations. Though we purposively selected facilities to reflect the different levels of public healthcare available in Nigeria, the small number of facilities and observations means that results cannot be generalized to the healthcare system. Data may be influenced by the Hawthorne effect, where HCW behaviour is influenced by the presence of the observer. However, we would expect this to result in a positive bias, where data would present higher rates of compliance than experienced on a day without observation. Lastly, though we minimized this through effective training of data collectors, it is plausible that some hygiene activities were not recorded by the data collector, especially given the stochastic nature of the moments of delivery, and this may negatively bias hand hygiene compliance findings. 

## 5. Conclusions

Reducing maternal and neonatal mortality will require targeted interventions to address infection risks in the critical window of birth and the first 48 hours of life. We must improve the quality of care women and babies receive in this period, and our study highlights that compliance with hand hygiene protocol is an acute stumbling block to achieving quality of care. Compliance levels were low across all actors even after facilities received external support on quality of care issues and hardware for handwashing, suggesting that improvement will require institutional change to challenge norms. Incorporating the WHO five moments for hand hygiene concept into trainings is recommended to ensure target behaviour is triggered at the appropriate time, as is the allocation of more time within training to hygiene. 

## Figures and Tables

**Table 1:** Hand hygiene categories used in analysis.

Hygiene Category	Definition
1	Hands washed with soap and new gloves applied, no potential recontamination observed
2	Hands washed with soap, but no gloves are worn, no potential recontamination observed
3	Gloves are changed but HWWS is not observed, no potential recontamination observed
4	No hand hygiene actions taken following observed invalidation of aseptic technique (contact with intact skin of the woman/new born or surroundings)
5	No hand hygiene actions taken following observed potential recontamination from high risk exposure (contact with another patient, bodily fluids, mucous membranes, non-intact skin, clinical waste or faeces)

**Table 2:** Hygiene category (using simplified hygiene scoring) during mother-specific procedures requiring aseptic technique during labour and delivery by provider type, facility type, state, and shift pattern.

		Hygiene Risk Category			Somers’ D Clustered by Actor; *p*-Value (Confidence Interval)
	*n*	Procedures Conducted in Category: Risky	Procedures Conducted in Category: Inadequate	Procedures Conducted in Category: Compliant	
**All events**	201	120 (60%)	74 (37%)	7(3%)	
**Provider Type**					
Nurse/Midwife	142	84 (60%)	52 (37%)	6 (4%)	ref
Doctor	58	35 (60%)	22 (38%)	1 (2%)	−0.02; *p* = 0.778 (−0.17 to 0.13)
**Facility Type**					
Primary	87	53 (61%)	30 (35%)	4 (5%)	ref
Secondary	61	35 (57%)	24 (39%)	2 (3%)	−0.01; *p* = 0.954 (−0.19 to 0.18)
Tertiary	53	32 (60%)	20 (38%)	1 (2%)	−0.04; *p* = 0.752 (−0.25 to 0.18)
**State**					
Ebonyi	119	73 (61%)	41 (35%)	5 (4%)	ref
Kogi	82	47 (57%)	33 (40%)	2 (3%)	0.32; *p* = 0.674 (−0.12 to 0.18)
**Shift**					
Morning	79	37 (47%)	38 (48%)	4 (5%)	ref
Afternoon	47	31 (66%)	15 (32%)	1 (2%)	0.19; *p* = 0.034 (−0.37 to 0.02)
Night	75	52 (69%)	21(28%)	2 (3%)	−0.23; *p* = 0.008 (−0.04 to −0.06)

**Table 3:** Hygiene risk category during all procedures requiring aseptic technique, including: vaginal examinations, insertion of urine catheter or IV cannula, artificial rupture of the membranes, manual removal of placenta or blood clots, and suturing of the perineum.

Hygiene Risk Category	All Procedures Requiring Aseptic Technique		Mother-Specific Procedures Requiring Aseptic Technique		Neonate-Specific Procedures Requiring Aseptic Technique	
	*N*	%	*N*	%	*N*	%
1. Hands washed with soap and gloves changed	7	3%	7	4%	0	0%
2. Hands washed with soap (no gloves applied)	7	3%	5	2%	2	1%
3. Gloves changed (no handwashing with soap)	70	27%	68	34%	2	4%
4. No hand hygiene actions taken following observed invalidation of aseptic technique *	104	41%	57	28%	47	85%
5. No hand hygiene actions taken following higher risk exposure **	68	27%	63	31%	5	9%
**Total**	**256**		**201**		**55**	

* Contact with intact skin of the woman/newborn or surroundings. ** Contact with another patient, bodily fluids, mucous membranes, non-intact skin, clinical waste or faeces.

## Supplementary Materials

The following are available online at https://www.mdpi.com/1660-4601/16/7/1301/s1. Supplemental Information 1: Further Information about the MCSP program; Table S1: Hygiene categories for maternal-specific procedures requiring aseptic technique during labour and delivery; Table S2: Hygiene categories for neonate-specific procedures requiring aseptic technique during labour and delivery.
