# Supplementary material for: Hygiene During Childbirth: An Observational Study to Understand Infection Risk in Healthcare Facilities in Kogi and Ebonyi States, Nigeria
Source: Int J Environ Res Public Health. 2019 Apr 11;16(7):1301. doi: 10.3390/ijerph16071301 (PMC6479678; doi:10.3390/ijerph16071301)
Supplement: Supplementary file 1 [file ijerph-16-01301-s001.pdf]

International Journal of
Environmental Research and Public Health

MDPI

Supplementary Materials

Hygiene During Childbirth: An Observational Study to Understand Infection Risk in Healthcare Facilities in Kogi and Ebonyi States, Nigeria

Helen Buxton [1], Erin Flynn [2], Olutunde Oluyinka [3], Oliver Cumming [1], Joanna Esteves Mills [1], Tess Shiras [1], Stephen Sara [4] and Robert Dreibelbis [1,*]

1 London School of Hygiene and Tropical Medicine, Disease Control Department, London WC1E 7HT, United Kingdom; Helen.Buxton1@lshtm.ac.uk (H.B.); Oliver.Cumming@lshtm.ac.uk (O.C.); Joanna.EstevesMills@lshtm.ac.uk (J.E.M.); tess.shiras@gmail.com (T.S.)
2 Infection and Immunity, South Australian Health and Medical Research Institute, Adelaide 5000, Australia; Erin.Flynn@sahmri.com (E.F.);
3 Maternal and Child Survival Program (MCSP)/Save the Children Nigeria, Abuja, Nigeria; olutundemma@gmail.com
4 Maternal and Child Survival Program (MCSP)/ Save the Children US, Washington, DC 20036, USA; ssara@savechildren.org
* Correspondence: Robert.Dreibelbis@lshtm.ac.uk; Tel.: +44 (0) 20 7636 8636

Supplemental Information 1: MCSP further information

All facilities included in the study have received support from the United States Agency for International Development (USAID) funded Maternal and Child Survival Program (MCSP) to improve quality of care. MCSP/Nigeria launched in 2014 and focused on strengthening national level Maternal Newborn and Child Health (MNCH) policy and improving the quality and utilization of services in public and faith-based facilities in Kogi and Ebonyi states.

Trainings delivered through the MCSP programme include basic and comprehensive emergency and newborn care, essential newborn care, and quality of care (BEmONC, CEmONC, ENCC, QoC). Hand hygiene has been integrated into all these trainings, though it is not the primary focus; for example, in the ENCC training, only ten minutes was set aside for hand hygiene and focuses purely on demonstration of when to wash hands with no time for skills practice. The use of alcohol-based hand sanitizers is also mentioned in trainings.

In the six months prior to study observations all facilities commenced quality improvement (QI) programming which included an initial IPC training and quarterly follow-up in facilities (5 HCFs began QI in Dec 2016 and 1 HCF began in June 2017). Content covered in training corresponded to key point-of-care domains: routine cleaning, waste management, linen management, patient care equipment, personal protective equipment (PPE), gloves, prevention of needle-stick injuries and hand hygiene. Training on hand hygiene was predominantly focused on technique and did not integrate messaging around the WHO five moments of hand hygiene. MCSP worked with partners such as the state Ministry of Health, local government authorities (LGAs) and professional associations to step-up and institutionalize quarterly integrated supportive supervision visits to health facilities and LGAs in both states. During these visits the frontline facility staff were supported to develop an action plan for addressing the gaps identified during the visits especially on how to ensure availability of essential medicines such as Chlorohexidine for umbilical cord care, dispersible Amoxicillin for acute respiratory and possible severe bacterial infections (PSBI). Hygiene and IPC were also discussed during supervisory visits if a need was identified.

Facilities were also provided with basic hygiene supplies, including; handwashing stations, soap, personal protective supplies, disinfectant, and veronica buckets (a plastic bucket with a tap underneath for hand washing) for use in the delivery room

Table S1. hygiene category for mother specific procedures requiring aseptic technique during labour and delivery.

Hygiene risk category	All mother-specific procedures requiring aseptic technique	Vaginal Examination	Insertion of urine catheter	Insertion of IV cannula	Artificial rupture of membranes	Manual removal of placenta or blood clots	Suturing of perineum
1. Hands washed with soap and gloves changed	7 (4%)	6 (5%)	0	0	0	0	0
2. Hands washed with soap (no gloves applied)	5 (3%)	3 (2%)	1 (3%)	0	1 (6%)	1 (9%)	0
3.Gloves changed (no hand washing with soap)	69 (34%)	54 (45%)	6 (19%)	4 (40%)	2 (12%)	1 (9%)	2 (18%)
4. No hand hygiene action taken following observed invalidation of aseptic technique*	57 (28%)	37 (31%)	8 (26%)	3 (30%)	2 (18%)	1 (9%)	5 (45%)
5. No hand hygiene action taken higher risk exposure**	63 (31%)	21 (17%)	16 (52%)	3 (30%)	11 (65%)	8 (74%)	4 (37%)
Total	201	121	31	10	17	11	11

*contact with intact skin of the woman/newborn or surroundings

** contact with another patient, bodily fluids, mucous membranes, non-intact skin, clinical waste or feces

Table S2. Hygiene categories during neonate-specific procedures requiring aseptic technique during labour and delivery.

Hygiene risk category	All contact with cord	Clamping/tying of cord	Contact with cord stump
1. Hands washed with soap and gloves changed	0	0	0
2. Hands washed with soap (no gloves applied)	2 (4%)	0	2 (11%)
3.Gloves changed (no hand washing with soap)	1 (2%)	0	1 (5%)
4. No hand hygiene action taken following observed invalidation of aseptic technique*	47 (85%)	35 (97%)	12 (63%)
5. No hand hygiene action taken higher risk exposure**	5 (9%)	1 (3%)	4 (21%)
	55	36	19

*contact with intact skin of the woman/newborn or surroundings

** contact with another patient, bodily fluids, mucous membranes, non-intact skin, clinical waste or feces

© 2019 by the authors. Submitted for possible open access publication under the terms and conditions of the Creative Commons Attribution (CC BY) license (http://creativecommons.org/licenses/by/4.0/).
